# Controlled Substance Use and Clinical Outcomes of Elderly Patients After a Fall

**DOI:** 10.7759/cureus.22356

**Published:** 2022-02-18

**Authors:** Lauren B Gammel, Matthew Leonard, Hannah Wheeler, Ha Linh, Bracken Burns

**Affiliations:** 1 General Surgery, East Tennessee State University-Quillen College of Medicine, Johnson City, USA; 2 Trauma, Johnson City Medical Center, Johnson City, USA; 3 Public Health, East Tennessee State University-Quillen College of Medicine, Johnson City, USA; 4 Surgery, East Tennessee State University-Quillen College of Medicine, Johnson City, USA

**Keywords:** fall, outcomes, narcotics, controlled substance, geriatric, trauma

## Abstract

Controlled substance use, particularly among the rising elderly population, places these patients at a much higher risk of falls, injuries, and hospitalization. This study examines the association between preinjury controlled substance prescription and clinical outcomes of older adults after a ground-level fall. A total of 5,930 patients were included. Their home medication list was analyzed to record active opioids, benzodiazepines, narcotics, or other substances defined as controlled according to the Drug Enforcement Agency. Almost half (45%) of the patients were taking controlled substances. Sixty-seven percent of those were females. Total hospital days, Injury Severity Score (ISS), and mortality outcomes were not significantly different between groups. However, intensive care unit (ICU) days, days on mechanical ventilation (MV), and discharge destination were significantly different for patients taking controlled substances versus those not taking controlled substances. Patients taking controlled substances were more likely to be discharged to short- and long-term care facilities versus patients not taking controlled substances (P≤0.001).

## Introduction

The United States is currently experiencing an opioid crisis. In 2016, there were more than 42,000 opioid-related overdose deaths in the United States, 115 deaths per day. Nearly, 7,000 of these deaths occurred in five states in the Appalachian region, Alabama, Kentucky, Ohio, Tennessee, and West Virginia [1]. Our center is located in Northeast Tennessee and the Southern Appalachia region. Polypharmacy and controlled substance use among the elderly patient population is of increasing concern. It has been reported that there are 94.4 opioid prescriptions per 100 persons in this population [2].

The prevalence of controlled substance use among trauma patients is thought to be high. A 2013 study in a Kentucky trauma center reported that 20% of their trauma patient population was taking one or more controlled substance before their injury [3]. Previous work from our institution showed a 29.9% positive rate of controlled substance use among trauma patients [4]. Falls are the leading cause of fatal injury and the most common cause of nonfatal trauma-related hospital admissions among older adults nationally [5]. The leading cause of trauma at our institution is a ground-level fall, which is more common among the elderly. We have observed a significant amount of preinjury controlled substance prescriptions among our elderly trauma patient population.

Numerous studies have attempted to define the impact of opioid use, specifically on patient outcomes. Multiple studies have shown controlled substance use to be associated with higher rates of mechanical ventilation (MV) and more time in the intensive care unit (ICU) [3,6,7]. Two recent reports specifically studied these outcomes in the trauma patient population with no consideration given to patient age [6,7]. Other studies showing this type of outcome correlation have been done in medical and surgical ICU patients without looking specifically at age or previous trauma. In geriatric patients, falls account for the most common cause of traumatic brain injury, death, and injury overall [8]. In 2015 alone, the total national medical costs for falls totaled more than $50 billion [9]. Another study in 2015 concluded that preinjury opioid users in the general trauma population are more likely to have presented with a fall as their mechanism of injury [10]. The rate of prescribed controlled substances (especially among geriatric patients, who are more likely to suffer a fall) is of persistent concern both regionally in Appalachia and nationally.

The Geriatric Trauma Committee describes frailty, age-specific alterations in physiologic response to shock, delirium, anticoagulants, and other medications as major factors leading to poor outcomes in geriatric trauma patients [8]. The elderly trauma patient population continues to grow rapidly. This is a vulnerable patient population with unique problems complicating the spectrum of their post-trauma care. Due to the significant burden of this injury mechanism on our elderly patients and our healthcare system overall, we directed our study toward this increasingly important subset of trauma patients.

The objectives of the study were to examine the association between controlled substance use, risk, and clinical outcomes with mortality and discharge disposition of fall-related injury in older adults. We hypothesized that patients who fell with prescriptions filled for controlled substances were more likely to experience longer length of hospital stay, ICU stay, and days requiring MV.

## Materials and methods

This is a single-center retrospective review using data from the trauma registry at the Johnson City Medical Center in Johnson City, Tennessee. We included all patients with ground-level falls (GLFs) aged 60 years and older during a seven-year period between January 1, 2011, and December 31, 2017. The Institutional Review Board (IRB) approved waiver of consent. Patient home medication lists were analyzed by pharmacy reconciliation. Active opioids, benzodiazepines, narcotics, or other substances defined within schedules one through five according to the Drug Enforcement Agency controlled substance schedules 2019 revision were the focus of this study.

Statistical significance was set at P<0.05. Chi square analysis was used to test significance for discharge destination to short- and long-term care facilities, mortality, and gender. T tests were performed to identify any significance between age, total hospital days, intensive care unit days, mechanical ventilation, and injury severity score. Logistic regression was used to determine if the number of controlled substance on home medication lists showed any significant increase in mortality. Statistical tests were completed using Excel v2108 (Microsoft, USA) and JASP v0.14.1.0 software.

## Results

There were a total of 5,930 patients in this study. A total of 3,259 (55%) were not taking a controlled substance, and 2,671 (45%) were taking one or more controlled substances. Of the 2,671 patients taking controlled substances, 1,646 (61%) patients were on one controlled substance, 835 (31%) patients were on two controlled substances, and 190 (8%) patients were on three or more controlled substances. Sixty-seven percent of females were taking controlled substances, while males were numbered at 33%. Females were more common in every group. Combination hydrocodone-acetaminophen was the most common controlled substance (686), followed by alprazolam (576) and lorazepam (535). A total of 56 controlled substances or combination of controlled substances were observed on patients’ home medication list. A comprehensive table of all controlled substances found on patients' home medication lists is shown in the Appendix, Table 3. The top 10 controlled substances found on patients' home medication list can be seen in Table 1. 

**Table 1:** Top 10 controlled substances found on patients' home medication list.

Name of Substance	Number Found on Patients Home Medication Lists
Hydrocodone with acetaminophen	686
Alprazolam	576
Lorazepam	535
Tramadol	371
Hydrocodone	297
Clonazepam	229
Zolpidem	203
Oxycodone with acetaminophen	176
Oxycodone	151
Diazepam	112

There was no difference in age between the two study groups. In fact, the mean age was identical for both groups. Injury Severity Score (ISS), gender, and hospital length of stay (LOS) also showed no difference and were nearly identical. Patients were more likely to experience longer ICU days (4) taking controlled substances versus taking no substances (3) (P=0.01). Patients were also more likely to experience longer mechanical ventilator days (MVD) (7) taking controlled substances versus taking no substances (4), (P=0.02) (Table 2). Patients taking controlled substances were more likely to be discharged to short- and long-term care facilities versus patients not taking controlled substances (P=0.001) (Table 2). 

**Table 2:** Descriptive statistics for demographics and discharge disposition. IQR: interquartile range.

	No Controlled Substances	Controlled Substances	P Value
Age			
N	3,259	2,368	0.279
Mean	76	76	
Standard deviation	8.323	8.215	
Injury Severity Score			
N	3,259	2,665	0.928
Mean	9	9	
Standard deviation	5.816	5.874	
Gender			
Male N (%)	1,069 (55%)	876 (45%)	0.997
Female	3,259 (55%)	2,671 (45%)	
Total hospital days			
N	3,259	2,671	0.314
Mean	5	5	
Median/IQR hospital days	4 IQR=3	4 IQR=3	
Standard deviation	5.000	5.018	
Total ICU days			
N	406	353	.012
Mean	3	4	
Standard deviation	3.070	5.416	
Total vent days			
N	87	90	0.028
Mean	4	7	
Standard deviation	5.125	8.716	
Discharge disposition			
Short- and long-term care facilities	(66%)	(70%)	<0.001
No extended facility	(34%)	(30%)	

Regression analysis showed that patients taking controlled substances had a weak positive correlation between ISS and MVD (P=0.04) and there was a moderate correlation between ICU, length of stay (LOS), and MVD (P≤0.001). Multiple logistic regression revealed that there was no significance in mortality between patients taking controlled substances versus those not taking them. However, further analysis showed that increased ISS (P≤0.001), increased ICU, LOS (P≤0.001), and increased age (P≤0.001) were all statistically significantly associated with increased mortality.

## Discussion

The use of controlled substances among trauma patients at our rural Level 1 Trauma Center in Northeast Tennessee had previously been reported as high as 29.9%, regardless of age [4]. We now report an alarmingly high prevalence of controlled substance prescription among the elderly GLF patients in our region (45%).

Previously, the published literature has shown negative clinical outcomes associated with preinjury opioid or controlled substance use in the trauma population including increased hospital length of stay, increased ICU length of stay, increased need for MV, and in-hospital mortality [3,7,10,11]. Only one of these studies has been aimed directly toward the elderly trauma patient population [8]. Additionally, there are very limited data comparing outcomes based on a similar injury mechanism such as a ground-level fall.

Our results show that the geriatric GLF patient’s use of controlled substances is associated with increased average length of ICU stay, increased average days requiring MV, and an increased likelihood of requiring posthospital short- and long-term facility placement upon discharge. In contrast to some current publicized literature, we found no significance between mortality or ISS and preinjury controlled substance use. Based on these findings, it does not appear that preinjury controlled substance prescription places geriatric patients at any higher risk for increased ISS or mortality but did portend worse outcomes following equivalent injury mechanisms. Additionally, other studies have shown that the use of multiple controlled substances by the same patient resulted in longer hospital and longer ICU stays, though we did not have similar findings [7]. When comparing patients taking one versus two or three controlled substances, there was no statistically significant difference in these parameters in this study.

We recognize that there are limitations to this study. Due to the retrospective nature of this analysis, we were unable to evaluate or analyze current or active use of controlled substances. Additionally, we were unable to ascertain the indication of these prescription medications. This information could have provided an indicator of high-risk status, rather than a causal relationship of these prescription drugs. Nonprescription drug intake was also unfortunately unable to be reviewed in the retrospective process, though future investigations with this purpose in mind would be intriguing. Additionally, we did not observe the timeframe from when a patient was prescribed their medication/s until the time of their accident. We feel that further evaluation of patients’ outcomes with regard to specific injury characteristics or the anatomic area of injury (i.e., the chest or intracranial) could prove to be an interesting and beneficial effort in the future.

At our institution, this information will be shared with the injury prevention coordinator who works with seniors regularly on fall prevention. These results have important clinical implications for the trauma surgeon as well as all physicians prescribing controlled substances as we attempt to improve geriatric healthcare. Our data and the preventative implications of our results will be communicated across educational platforms aimed toward primary care physicians within our hospital system. Early recognition of these higher-risk elderly patients and anticipation of their unique needs with strategic protocols will hopefully improve our clinical outcomes and resource allocation.

Evidence of the correlation between controlled substance use and clinical outcomes of the geriatric trauma patient allows clinicians to identify patients most at risk for adverse outcomes and to plan management accordingly. This knowledge could lead to improved triage protocols, risk stratification, and subsequent management of the injured elderly patient under the physiologic influences of controlled substances. Further investigations should be directed at improving ICU care and disposition planning of geriatric trauma patients taking controlled substances preinjury.

## Conclusions

Ground-level fall patients aged 60 years and older who were prescribed controlled substances preinjury experienced increased ICU stay, mechanical ventilation days, and an increased likelihood of requiring posthospital short- and long-term facility placement upon discharge when compared to those not taking controlled substances. Preinjury controlled substance prescription was not associated with geriatric patients at higher risk for increased ISS or mortality but is associated with longer ICU days, MV days, and discharge to short- and long-term care facilities following similar injury mechanisms.

## Appendices

Appendix

**Table 3:** Controlled substances found on patients' home medication list.

Name of Controlled Substance
Alprazolam
Atropine
Belladonna
Buprenorphine with naloxone
Carisoprodol
Chlordiazepoxide
Clonazepam
Clorazepate
Clorazepate dipotassium
Codeine
Codeine with acetaminophen
Codeine with guaifenesin
Codeine with Tylenol
Codeine with promethazine
Diazepam
Dihydrocodeine
Diphenoxylate with atropine
Dronabinol
Eszopiclone
Fentanyl
Fentanyl patch
Fiorinal
Flurazepam
Hydrocodone
Hydrocodone bitartrate
Hydrocodone with acetaminophen
Hydrocodone with chlorpheniramine
Hydrocodone with ibuprofen
Hydromorphone
Lacosamide
Lisdexamfetamine
Lorazepam
Meperidine
Meprobamate
Methadone
Methylphenidate
Modafinil
Morphine
Oxazepam
Oxycodone
Oxycodone with acetaminophen
Oxymorphone
Pentazocine with naloxone
Perampanel
Phenobarbital
Phentermine
Pregabalin
Propoxyphene with acetaminophen
Temazepam
Testosterone
Theophylline
Topiramate
Tramadol
Tramadol with acetaminophen
Trazodone
Triazolam
Zaleplon
Zolpidem
